# Combining ASBT inhibitor and FGF15 treatments enhances therapeutic efficacy against cholangiopathy in female but not male *Cyp2c70* KO mice

**DOI:** 10.1016/j.jlr.2023.100340

**Published:** 2023-02-03

**Authors:** Mohammad Nazmul Hasan, Jianglei Chen, David Matye, Huaiwen Wang, Wenyi Luo, Lijie Gu, Yung Dai Clayton, Yanhong Du, Tiangang Li

**Affiliations:** 1 Harold Hamm Diabetes Center, Department of Physiology, University of Oklahoma Health Sciences Center, Oklahoma City, OK, USA; 2 Laboratory for Molecular Biology and Cytometry Research, University of Oklahoma Health Sciences Center, Oklahoma City, OK, USA; 3 Department of Pathology, University of Oklahoma Health Sciences Center, Oklahoma City, OK, USA

**Keywords:** bile acid, ASBT, CYP7A1, FXR, cholestasis, CYP2C70, 7α-HSDH, 7α-hydroxysteroid dehydrogenase, 7β-HSDH, 7β-hydroxysteroid dehydrogenase, ABST, apical sodium-dependent bile acid transporter, AAV, adeno-associated virus, CA, cholic acid, CDCA, chenodeoxycholic acid, DCA, deoxycholic acid, FGF15, fibroblast growth factor 15, FGF19, fibroblast growth factor 19, FXR, farnesoid x receptor, LCA, lithocholic acid, MCA, muricholic acid, OCA, obeticholic acid, OSTB, organic solute transporter, PXR, pregnane x receptor, SULT2A1, sulfotransferase 2A1, T-CA, tauro-conjugated cholic acid, T-CDCA, tauro-conjugated CDCA, T-DCA, auro-deoxycholic acid, T-LCA, tauro-lithocholic acid, T-UDCA, tauro-conjugated ursodeoxycholic acid, UDCA, ursodeoxycholic acid, ZO-1, Zonula Occludens-1

## Abstract

Therapeutic reduction of hydrophobic bile acids exposure is considered beneficial in cholestasis. The *Cyp2c70* KO mice lack hydrophilic muricholic acids and have a human-like hydrophobic bile acid pool resulting in hepatobiliary injury. This study investigates if combining an apical sodium-dependent bile acid transporter inhibitor GSK2330672 (GSK) and fibroblast growth factor-15 (FGF15) overexpression, via simultaneous inhibition of bile acid synthesis and gut bile acid uptake, achieves enhanced therapeutic efficacy in alleviating hepatobiliary injury in *Cyp2c70* KO mice. The effects of GSK, adeno-associated virus (AAV)-FGF15, and the combined treatment on bile acid metabolism and cholangiopathy were compared in *Cyp2c70* KO mice. In female *Cyp2c70* KO mice with more severe cholangiopathy than male *Cyp2c70* KO mice, the combined treatment was more effective in reversing portal inflammation, ductular reaction, and fibrosis than AAV-FGF15, while GSK was largely ineffective. The combined treatment reduced bile acid pool by ∼80% compared to ∼50% reduction by GSK or AAV-FGF15, and enriched tauro-conjugated ursodeoxycholic acid in the bile. Interestingly, the male *Cyp2c70* KO mice treated with AAV-FGF15 or GSK showed attenuated cholangiopathy and portal fibrosis but the combined treatment was ineffective despite reducing bile acid pool. Both male and female *Cyp2c70* KO mice showed impaired gut barrier integrity. AAV-FGF15 and the combined treatment, but not GSK, reduced gut exposure to lithocholic acid and improved gut barrier function. In conclusion, the combined treatment improved therapeutic efficacy against cholangiopathy than either single treatment in the female but not male *Cyp2c70* KO mice by reducing bile acid pool size and hydrophobicity.

Bile acids are synthesized from cholesterol in hepatocytes and circulate between the liver and intestine in a process called the enterohepatic circulation (1). By doing so, bile acids act as physiological detergents to solubilize cholesterol in the bile and emulsify dietary lipids and fat-soluble vitamins in the small intestine. Bile acids also regulate a wide range of cellular pathways by activating nuclear receptors and cell surface receptors (1). In this regard, bile acid activated farnesoid x receptor (FXR) plays a key role in regulating bile acid homeostasis. Bile acids in hepatocytes activate FXR to feedback inhibit the transcription of the cholesterol 7α-hydroxylase (*CYP7A1*) gene, which encodes CYP7A1, the rate-limiting enzyme in de novo bile acid synthesis (2). Bile acids also activate intestinal FXR to induce mouse fibroblast growth factor 15 (FGF15) and human fibroblast growth factor 19 (FGF19), the ortholog of FGF15, which inhibit the *CYP7A1* gene and bile acid synthesis via a gut-liver endocrine axis (3). Mouse hepatocytes do not express FGF15, while human hepatocytes express FGF19, which is induced by hepatic FXR to inhibit CYP7A1 in an autocrine manner (4, 5). These liver and intestine bile acid-sensing mechanisms help to maintain a relatively constant bile acid pool and prevent intracellular bile acid accumulation.

Both genetic and acquired impairment of bile flow out of the liver causes cholestasis, a pathological condition associated with intrahepatic bile acid accumulation leading to liver injury, inflammation, and fibrosis (1). The hydrophilic bile acid, ursodeoxycholic acid (UDCA), and FXR agonist obeticholic acid (OCA) are approved therapies to treat a few forms of human cholestasis (6). However, therapeutic options are still limited for patients who do not adequately respond to these treatments. Because hydrophobic bile acid-induced damage to hepatocytes and bile duct epithelial cholangiocytes is a key pathogenic driver in cholestasis, FGF19 analogue that inhibits bile acid synthesis and apical sodium-dependent bile acid transporter (ASBT) inhibitor that blocks intestine bile acid reuptake have been tested as potential treatments for various forms of human cholestasis (7, 8, 9, 10, 11). Due to the bile acid-mediated feedback regulation of bile acid synthesis and transport in the liver and the intestine, inhibition of de novo bile acid synthesis and a smaller bile acid pool decreases fecal bile acid loss, while blocking intestinal bile acid uptake causes compensatory induction of de novo bile acid synthesis. These compensatory mechanisms are expected to limit the magnitude of bile acid pool reduction that can be achieved by FXR agonists, FGF19 analogues, or ASBT inhibitors. Recently, we reported that combining a gut restricted ASBT inhibitor GSK2330672 (GSK) with adeno-associated virus (AAV)-FGF15–mediated hepatocyte-specific FGF15 overexpression, which mimics FGF19 analogue therapy, effectively inhibited both hepatic bile acid synthesis and intestinal bile acid reuptake, which achieved a significantly higher degree of bile acid pool reduction than either single treatment in WT mice (12). We also showed that in obese mice with nonalcoholic steatohepatitis, this combined treatment decreased gut cholesterol and fatty acid absorption, obesity, and hepatic steatosis and fibrosis (12). This cotreatment draws close comparison to the U.S. Food and Drug Administration approved cholesterol lowering combination drug Vytorin (simvastatin/ezetimibe), in which simvastatin inhibits de novo cholesterol synthesis and ezetimibe inhibits intestinal cholesterol absorption. The recently completed clinical trial reports that by targeting both hepatic and intestinal sources of cholesterol Vytorin is significantly more effective in achieving the more stringent low density lipoprotein-cholesterol lowering goal in type-2 diabetes patients and decreasing cardiovascular events and mortality in the high-risk population (13).

Because patients with various forms of genetic and acquired cholestasis may presumably benefit from a higher degree of bile acid pool reduction that may not be achieved by a monotherapy, we further investigated the therapeutic benefits of combining GSK and AAV-FGF15 in alleviating hepatobiliary bile acid toxicity in the *Cyp2c70* KO mice that were generated recently in our lab. CYP2C70 mediates the synthesis of muricholic acids (MCAs) primarily from chenodeoxycholic acid (CDCA) in mice and is responsible for the species difference of primary bile acids in humans and mice (14, 15, 16, 17). In comparison to human bile acid pool, the significantly less hydrophobic bile acid pool due to the presence of MCAs in mice has been a limitation of investigating bile acid-induced hepatobiliary toxicity and the pathological impact of altered bile acid composition observed in WT mice cannot be extrapolated to humans (18). The bile of the *Cyp2c70* KO mice contains primarily hydrophobic CDCA and cholic acid (CA), and these mice exhibit a human-like hydrophobic bile acid pool-induced hepatobiliary injury phenotype. This study revealed differential impact of GSK, AAV-FGF15, and the combined treatment on bile acid pool size and composition and the therapeutic efficacy toward cholangiopathy, portal fibrosis, and gut barrier integrity in *Cyp2c70* KO mice.

## MATERIALS AND METHODS

### Reagents

Aspartate aminotransferase and alanine aminotransferase assay kits were purchased from Pointe Scientific (Canton. MI). GSK2330672 was purchased from MedChemExpress LLC (Monmouth Junction, NJ). Bile acid assay kit was purchased from Diazyme Laboratories (Poway, CA). AAV-FGF15 (under the control of an albumin promoter) was purchased from GeneCopoeia, Inc. (Rockville, MD). AAV-Null was purchased from Vector Biolabs Inc. (Malvern, PA). F4/80 antibody (Cat #. 70,076) was purchased from Cell Signaling Technology (Danvers, MA). Cytokeratin-19 antibody (ab52625) was purchased from Abcam (Waltham, MA). ZO-1 antibody (PA5-28858) was purchased from Thermo Fisher Scientific (Grand Island, NY).CDCA-d4 MaxSpec® Standard (CDCA-d4, No. 31366), tauro-conjugated CDCA-d4 MaxSpec® Standard (T-CDCA-d4, No. 31362), UDCA-d4 MaxSpec® Standard (UDCA-d4, No. 31368), and Tauro-conjugated UDCA-d4 MaxSpec® Standard (T-UDCA-d4, No. 31564) were purchased from Caymen Chemical Company (Ann Arbor, MI). FITC-dextran (MW:3000–5000) was purchased from Sigma Aldrich (St. Louis, MO).

### Mice

The *Cyp2c70* KO mice with Exon 4 deletion were generated by Clustered Regularly Interspaced Short Palindromic Repeats/Cas-mediated genome engineering. Cas9 and gRNA (gRNA1 matching reverse strand of the *Cyp2c70* gene: CTCTCATCACGGCACAACTTAGG; gRNA2 matching forward strand of the *Cyp2c70* gene: TAAAGAGGCCACTAAATTGCTGG) were coinjected into fertilized eggs of C57BL/6J mice. One F0 male founder mouse was identified by PCR followed by sequencing analysis to confirm deletion of an 888 bp genome region containing the entire Exon 4. This F0 founder was bred to a WT C57BL/6J mouse to obtain 3 F1 *Cyp2c70* ± females confirming germline transmission. Further breeding of the F1 females with WT C57BL/6J mice (the Jackson Lab, Bar Harbor, ME) yielded WT mice and *Cyp2c70* ± mice. Subsequent breeding of the *Cyp2c70* ± offspring yielded WT mice and *Cyp2c70*−/− mice (*Cyp2c70* KO). The *Cyp2c70* KO mice were then bred via KO x KO breeding scheme. The WT littermates were used to generate WT mice via WT x WT breeding scheme. The *Cyp2c70* KO pups weaned at 3 weeks of age showed significantly increased mortality, which was largely prevented when they were weaned at 4 weeks of age. Mice were housed in microisolator cages with Biofresh performance bedding (Pelleted cellulose) under 7 am−7 pm light cycle and 7 pm−7 am dark cycle. GSK was mixed with chow diet to achieve an estimated 5 mg/kg/day intake based on 5 g/day food intake by a 25 g mouse. AAV-Null or AAV-FGF15 was injected via tail vein at a dose of 1 ×10^11^ GC/mouse. Mice were euthanized following a 6 h fast from 9 am to 3 pm and tissues and blood samples were collected. To evaluate gut barrier function, mice under the described treatments for ∼3–4 weeks were fasted for ∼6 h starting at around 9 am. FITC-dextran was prepared in sterile PBS (60 mg/ml) and a single dose (600 mg/kg BW) was administered to each mouse via oral gavage. Blood was collected immediately before and at 1 h after FITC-dextran administration. FITC fluorescence in plasma samples was measured with a Tecan M200 PRO plate reader (excitation: 485 nm; emission: 530 nm). The fluorescent value at 0 h (background) was subtracted from the value at 1 h of the same mouse and the blood FITC-dextran concentration was calculated based on a standard curve. Animals received humane care according to the criteria outlined in the “Guide for the Care and Use of Laboratory Animals.” All animal protocols were approved by the Institutional Animal Care and Use Committee of the University of Oklahoma Health Sciences Center.

### Bile acid analysis: total bile acid measurement and LC-MS method

Bile acids were extracted from liver, whole gallbladder bile, whole small intestine with content, and dried feces in 90% ethanol. Briefly, solid tissues and feces were homogenized in 90% ethanol and incubated at 50°C overnight. After centrifugation, the clear supernatant was used for total bile acid measurement with a bile acid assay kit. To collect fecal samples, an individual mouse was placed in a jar briefly and fresh feces were collected. Bile acid pool was calculated as the total bile acids in liver, gallbladder, and small intestine. For LC-MS analysis of bile acid composition, ethanol extracts were dried and then resuspended in injection buffer containing 0.1% formic acid in 1:1 water: mixture of acetonitrile and methanol (1:1) and subsequently analyzed on a Thermo Fisher Scientific UltiMate 3000 UHPLC with a Waters Cortecs C18 column (Waters Acquity UPLC HSS T3 1.8 um, 2.1x150 mm, part No. 186003540) and a TSQ Quantis triple quadrupole mass spectrometer. The running condition is as follow: Solvent A: 0.1% formic acid; Solvent B: 0.1% formic acid in 1:1 methanol: acetonitrile. Flow rate: 0.3 ml/min. Gradient: 52%–90% B in 18 min, 90% to 52%B in 0.1 min, hold for 4 min. Run time: 22 min. TSQ Quantis triple quadrupole mass spectrometer Ion Mode: Ion Source Type: H-ESI; Spray Voltage: Static; Negative Ion (V): 2500; Sheath Gas (Arb): 50; Aux Gas (Arb): 10; Sweep Gas (Arb): 1; Ion Transfer Tube Temp (°C): 325; Vaporizer Temp (°C): 350; Polarity: Negative; Cycle Time (sec): 0.8. Other LC-MS parameters (Retention time, ion monitoring) are listed in supplemental Table S1. Standard curves for bile acids and internal standard glyco-CDCA-d4 (G-CDCA-d4) were generated with purified compounds and relative area under the curve was calculated. To measure T-CDCA-d4 metabolism in fecal slurry mixtures, fresh fecal sample was resuspended in a reaction buffer consisting of 10% PBS (pH=7.4), and 90% 3 mM sodium acetate (pH = 5.2) to a final suspension of 4 mg fecal sample/ml. T-CDCA-d4 was added to a final concentration of 20 μg/ml and the mixture was incubated at 37°C for 6 h. An equal amount of methanol was added and the mixture was incubated on ice for 1 h to precipitate protein. After centrifugation, an aliquot of the supernatant was vacuum dried and resuspended in injection buffer. LC-MS measurement of bile acids was performed as described above.

### Liver and intestine histology and immunohistochemistry

Liver tissues and colon tissues were fixed in 4% paraformaldehyde and paraffin embedded. H&E staining was performed with an automated stainer. Sirius Red staining was performed with Direct Red 80 solution (Sigma #365548, St. Louis, MO). For immunohistochemistry, paraffin embedded tissues were deparaffinized and rehydrated, and after antigen retrieval, 5 μm sections were incubated first with blocking buffer (5% BSA and 5% goat serum in PBS) for 1 h and then blocking buffer containing primary antibodies overnight at 4°C. After washing with PBS, the sections were incubated with secondary antibodies in SignalStain® Boost IHC Detection Reagent (Cell Signaling, #8114, Danvers, MA) for 1 h. Signal was then visualized using a DAB kit (Cell Signaling, #11724, Danvers, MA). The sections were counterstained with Hematoxylin. Images are acquired by using an EVOS M5000 imaging system (Thermo Fisher Scientific, Grand Island, NY). Quantification was performed with ImageJ Fiji software (https://fiji.sc).

### Real-time PCR

Total liver RNA was purified with Trizol (Sigma-Aldrich, St. Louis, MO). Reverse transcription was performed by using Oligo dT primer and SuperScript III reverse transcriptase (Thermo Fisher Scientific, Grand Island, NY). Real-time PCR was performed on a Bio-Rad CFX384 Real-time PCR system with iQ SYBR Green Supermix (Bio-rad, Hercules, CA). 18S was measured and used for normalization. The comparative CT method was used to determine the relative mRNA expression with the control group arbitrarily set as “1”.

### Statistical analysis

All results were expressed as mean ± SEM. One-way ANOVA or Student’s *t* test was used to calculate the *P*-value. A *P* < 0.05 was considered statistically significant.

## RESULTS

### *Cyp2c70* KO mice show human-like hydrophobic bile acid pool and cholangiopathy and portal fibrosis

**Fig. 1:**
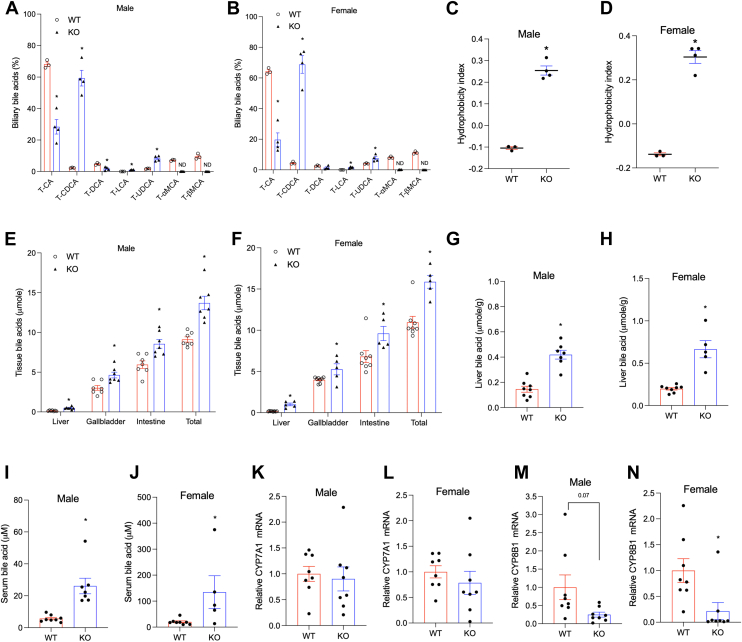
The *Cyp2c70* KO mice show enlarged hydrophobic bile acid pool than WT mice. WT and *Cyp2c70* KO mice at 16 weeks of age were fasted for 6 h from 9 am to 3 pm and euthanized. A, B, Gallbladder bile acid pool composition. N = 3–4. C, D, Hydrophobicity index calculated based on gallbladder bile acid composition. E, F, Tissue total bile acid content and total bile acid pool. N = 5–8. G, H, Liver total bile acid concentration (normalized to liver weight). N = 5–8. I, J, Serum bile acid concentration. N = 5–8. K–N. Relative liver mRNA expression. N = 8. All results are expressed as mean ± SEM. “∗” indicates statistical significance (*P* < 0.05, Student’s *t* test), versus WT. ND: not detectable. CK19, cytokeratin-19.

**Fig. 2:**
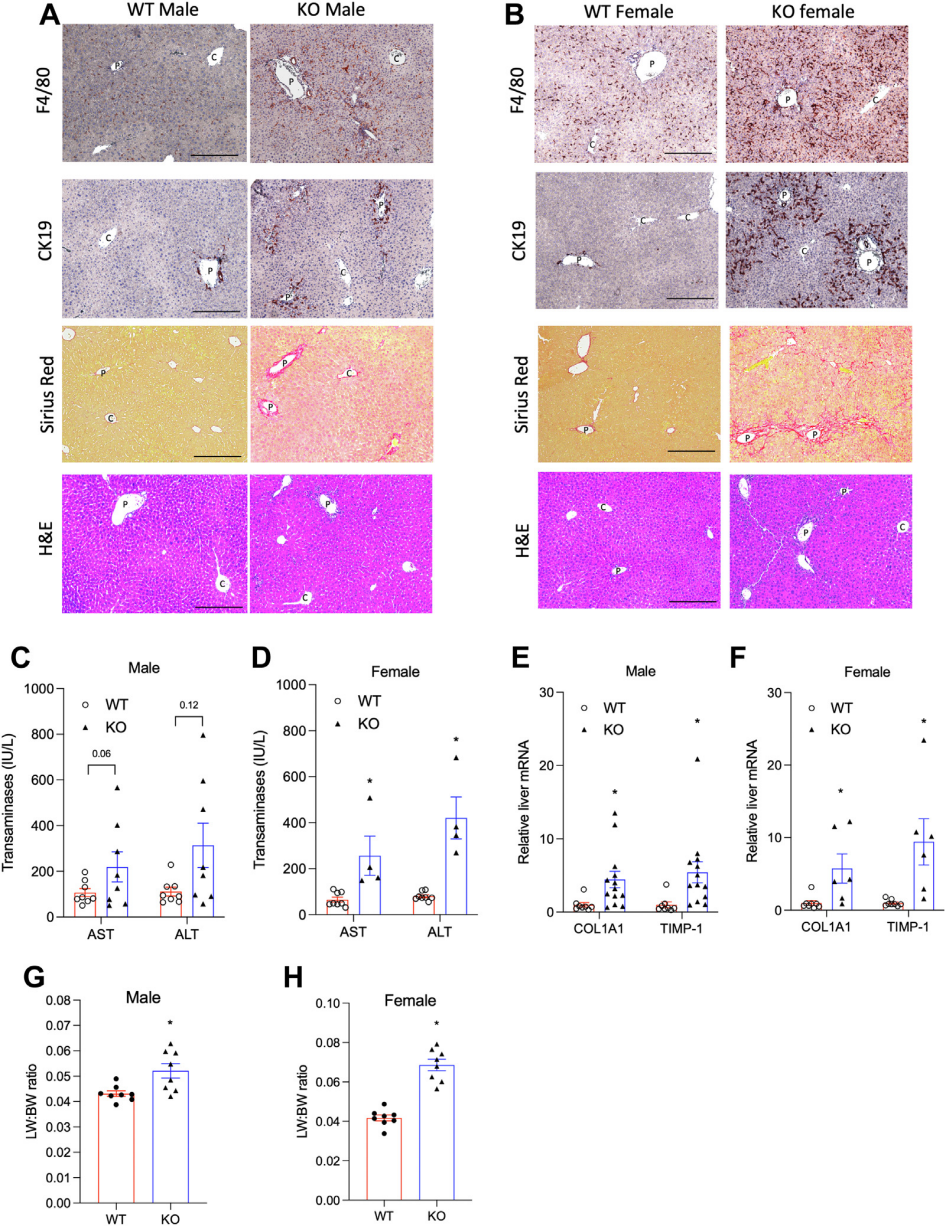
Portal inflammation, ductular reaction, and portal fibrosis in the male and female *Cyp2c70* KO and WT mice. WT and *Cyp2c70* KO mice at 16 weeks of age were fasted for 6 h from 9 am to 3 pm and euthanized. A, B, Representative images of immunohistochemistry of F4/80 stain and CK19 stain, and Sirius Red and H&E stain. p: portal vein; c: central vein. Scale bar = 250 um. C, D, Serum alanine aminotransferase (ALT) and aspartate aminotransferase (AST). N = 4–8. E, F, Relative liver mRNA expression. N = 6–13. G, H, Liver weight (LW) to body weight (BW) ratio. N = 8. All results are expressed as mean ± SEM. “∗” indicates statistical significance (*P* < 0.05, Student’s *t* test), versus WT. CK19, cytokeratin-19.

Tauro-conjugated MCAs (T-αMCA and T-βMCA) were detected in the gallbladder bile of the WT mice (Fig. 1A, B). It should be noted that because the bile acid panel analysis did not include T-ωMCA, the total tauro-conjugated MCA abundance was underestimated in the WT mice. Genetic deletion of the *Cyp2c70* gene resulted in complete absence of MCAs in the gallbladder bile of both male and female *Cyp2c70* KO mice (Figs. 1A, B and S1A, B). Due to the lack of CYP2C70-mediated conversion of CDCA to αMCA, the gallbladder bile acid pool in both 16 weeks old male and female *Cyp2c70* KO mice consisted primarily of ∼60–70% T-CDCA and ∼20–30% tauro-conjugated cholic acid (T-CA). Because CDCA can be converted to UDCA via 7-HO epimerization and CYP2C70 oxidizes UDCA to βMCA (18), the lack of CYP2C70 also resulted in ∼8% T-UDCA in the bile of the *Cyp2c70* KO mice, which was higher than the ∼2–4% of T-UDCA found in the bile of the WT mice (Fig. 1A, B). Consistent with increased T-CDCA, the bile acid pool of the *Cyp2c70* KO mice contained ∼ 1–1.5% of tauro-lithocholic acid (T-LCA) compared to ∼0.04% of T-LCA found in the gallbladder bile of the WT mice (Fig. 1A, B). Similarly, the bile acid pool of the *Cyp2c70* KO mice showed significantly lower tauro-deoxycholic acid (T-DCA) abundance (∼1–3%) than the WT mice, correlating with reduced T-CA abundance than the WT mice (Fig. 1A, B). The unconjugated bile acids collectively accounted for less than 1% of the overall bile acid pool and were not further analyzed. Due to these bile acid composition changes, the bile acid pool of the *Cyp2c70* KO mice showed a higher hydrophobicity index than that of the WT mice (Fig. 1C, D) (19). In addition to altered bile acid composition, the *Cyp2c70* KO mice showed significantly increased bile acid content in the liver, gallbladder, and small intestine, resulting in a ∼50% larger bile acid pool in both male and female *Cyp2c70* mice than the WT mice (Figs. 1E, F and S1C, D). Consistently, hepatic bile acid concentration was significantly increased by ∼3-fold in the *Cyp2c70* KO mice than the WT mice (Fig. 1G, H). Serum bile acid concentration was also significantly higher in the *Cyp2c70* KO mice than the WT mice (Fig. 1I, J). Despite a larger and more hydrophobic bile acid pool, the *Cyp2c70* KO mice showed similar liver CYP7A1 mRNA than the WT mice (Fig. 1K, L). It should be noted that reduced total bile acid pool and hepatic CYP7A1 expression in *Cyp2c70* KO mice compared to WT mice have been reported in independently generated *Cyp2c70* KO mice previously by other groups (16, 20). The mRNA of sterol 12α-hydroxylase (CYP8B1) was ∼80% lower in the *Cyp2c70* KO mice than the WT mice (Fig. 1M, N), which provided an explanation of reduced T-CA abundance in the *Cyp2c70* KO mice (Fig. 1A, B). At 16 weeks of age, both male and female *Cyp2c70* KO mice showed increased periportal inflammatory infiltration (F4/80 and H&E stain), ductular reaction (cytokeratin-19 stain), and portal fibrosis (Sirius Red stain) (Fig. 2A, B). Liver injury and portal fibrosis were consistent with elevated serum aspartate aminotransferase and alanine aminotransferase) (Fig. 2C, D), hepatic mRNA expression of COL1A1 and TIMP-1 (Fig. 2E, F), and increased liver weight to body weight ratio (Fig. 2G, H) in the *Cyp2c70* KO mice. However, the female *Cyp2c70* KO mice showed more severe inflammatory infiltration, ductular reaction and portal fibrosis than the male *Cyp2c70* KO mice (Fig. 2A, B). In summary, the *Cyp2c70* KO mice showed “human-like” hydrophobic bile acid pool composition resulting in female-predominant cholangiopathy and portal fibrosis.

### The combined ASBT inhibitor and AAV-FGF15 treatment attenuates biliary injury and portal fibrosis in the female *Cyp2c70* KO mice

**Fig. 3:**
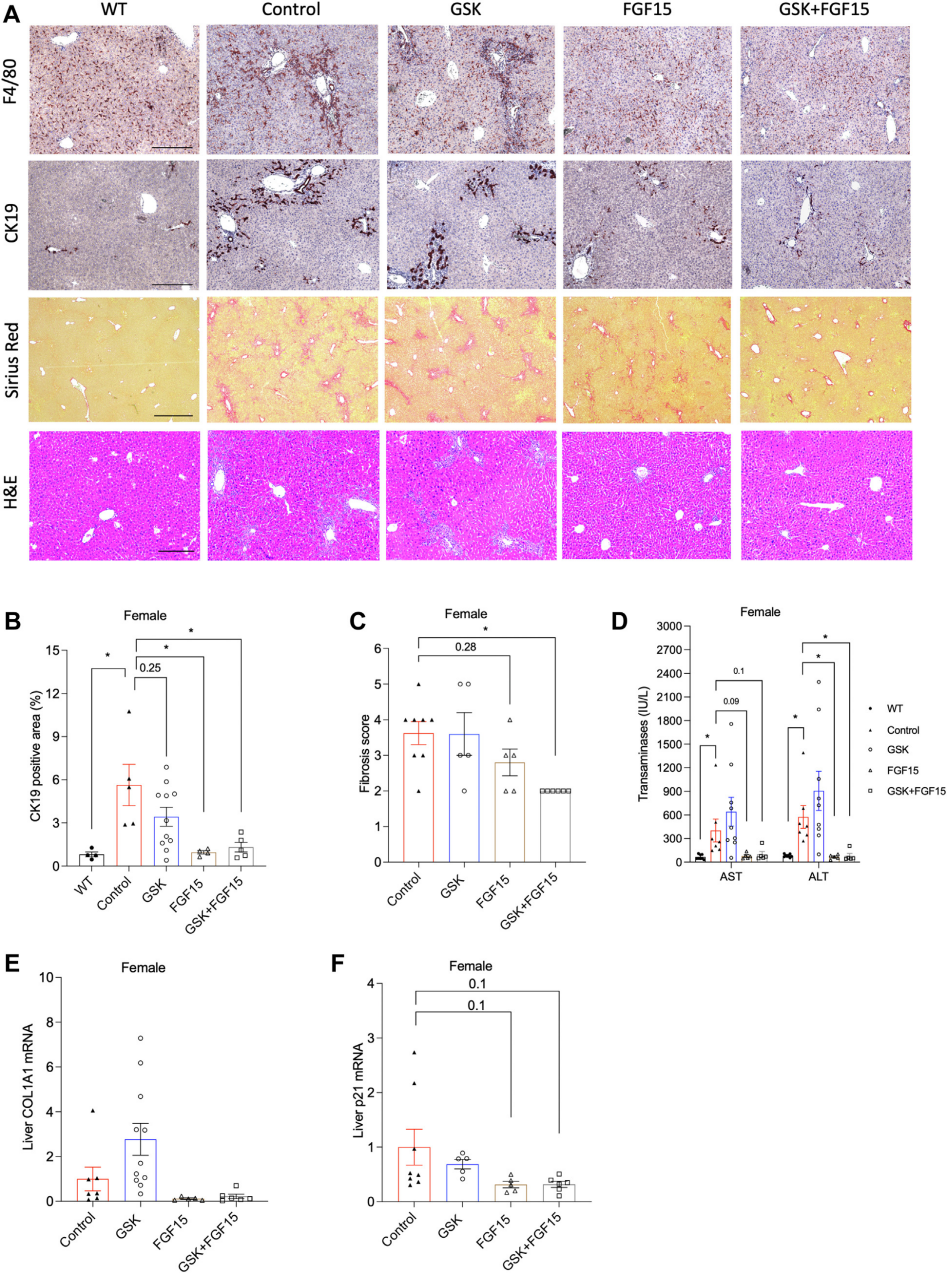
The therapeutic effects of GSK, AAV-FGF15, and the combined treatment in the female *Cyp2c70* KO mice. Female *Cyp2c70* KO mice at 12 weeks of age were injected with AAV-FGF15 (1 × 10^11^ GC/mouse) indicated as “FGF15”. Some mice were treated with GSK (5 mg/kg/day) indicated as “GSK”. The *Cyp2c70* KO mice in the “Control” group and the “GSK” group were injected with AAV-Null (1 × 10^11^ GC/mouse). After 4 weeks, mice were fasted for 6 h from 9 am to 3 pm and euthanized. A: Representative images of immunohistochemistry of F4/80 stain and CK19 stain, and Sirius Red and H&E stain. Scale bar = 250 um for F4/80, CK-19, and H&E; scale bar = 600 um for Sirius Red. Age matched WT mice were included for comparison. B: CK19 positive area per view was quantified with ImageJ software (https://fiji.sc). N = 4–11. C: Ishak fibrosis score. N = 5–8. D: Serum alanine aminotransferase (ALT) and aspartate aminotransferase (AST). N = 5–9. E, F, Relative liver mRNA expression. N = 5–8. All results are expressed as mean ± SEM. “∗” indicates statistical significance (*P* < 0.05, one way ANOVA and Dunnett’s post hoc test), versus Control. FGF15, fibroblast growth factor 15; CK19, cytokeratin-19.

We next compared the therapeutic benefits of a gut-restricted ASBT inhibitor GSK, AAV-FGF15, and the combination of the two treatments in modulating bile acid pool size and composition and liver injury in the *Cyp2c70* KO mice. In the female *Cyp2c70* KO mice with more severe liver injury, after 4 weeks of treatment initiated when mice were 12 weeks of age, the GSK treatment was largely ineffective in alleviating portal inflammation, ductular reaction, or fibrosis (Fig. 3A–C). The AAV-FGF15 treatment significantly decreased portal inflammatory infiltration and ductular reaction but was less effective in reversing portal fibrosis (Fig. 3A–C). In comparison, the combined treatment largely decreased portal inflammation, ductular reaction, and portal fibrosis (Fig. 3A–C). Consistent with these liver histopathological improvements, serum transaminases were reduced by AAV-FGF15 and the combined treatment but was not altered by the GSK treatment (Fig. 3D). The mRNA expression of liver fibrogenesis marker Collagen, type I, α1 (COL1A1) trended lower in the AAV-FGF15 treatment and the combined treatment groups, but not the GSK treatment group, although these changes did not reach statistical significance due to relatively large variations (Fig. 3E). Cellular senescence has recently been identified as a hallmark feature of biliary injury (21). GSK treatment did not affect liver p21 mRNA expression, while both the AAV-FGF15 treatment and the combined treatment reduced liver p21 mRNA by ∼70% (Fig. 3F), which closely correlated with reduced inflammatory infiltration and ductular reaction in these mice. In summary, both the AAV-FGF15 treatment and the combined treatment decreased portal inflammation and ductular reaction but only the combined treatment achieved reversal of portal fibrosis in the female *Cyp2c70* KO mice.

### The combined ASBT inhibitor and AAV-FGF15 treatment resulted in marked bile acid pool reduction coupled with T-UDCA enrichment in the female *Cyp2c70* KO mice

**Fig. 4:**
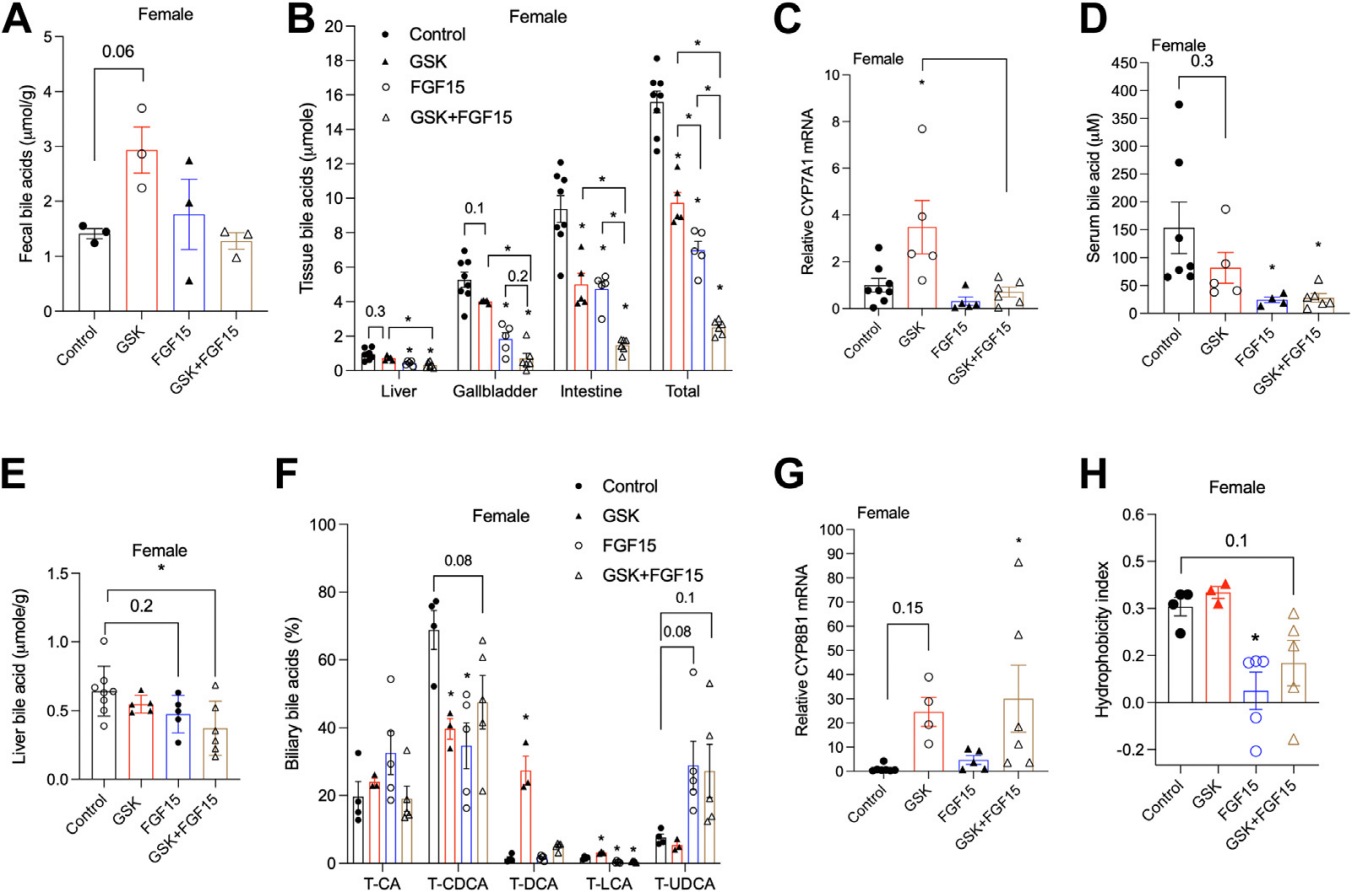
The effects of GSK, AAV-FGF15, and GSK+AAV-FGF15 treatment on bile acid metabolism in the female *Cyp2c70* KO mice. Female *Cyp2c70* KO mice at 12 weeks of age were injected with AAV-FGF15 (1 × 10^11^ GC/mouse) indicated as “FGF15”. Some mice were treated with GSK (5 mg/kg/day) indicated as “GSK”. Mice in the “Control” group and the “GSK” group were injected with AAV-Null (1 × 10^11^ GC/mouse). After 4 weeks, mice were fasted for 6 h from 9am to 3 pm and euthanized. A: Fecal total bile acids after 2 weeks of various treatments. N= 3. B: Tissue total bile acid content and total bile acid pool. N = 5–8. C: Relative liver mRNA expression. N = 5–8. D: Serum bile acid concentration. N = 4–7. E: Liver bile acid normalized to liver weight. N = 5–8. F: Gallbladder bile acid composition. N = 3–5. G|G, Relative liver mRNA expression. N = 4–7. H: Hydrophobicity index calculated based on gallbladder bile acid composition. N = 3–5. All results are expressed as mean ± SEM. “∗” indicates statistical significance (*P* < 0.05, one way ANOVA and Dunnett’s post hoc test), comparison is either versus “Control” or as indicated. FGF15, fibroblast growth factor 15.

To understand the underlying mechanisms associated with the observed therapeutic effects, we next studied bile acid metabolism in these mice. Fecal bile acid measurements revealed that the GSK treatment increased fecal bile acid loss by ∼2-fold after 2 weeks treatment, the AAV-FGF15 treatment did not alter fecal bile acid excretion after 2 weeks but its presence in the combined treatment prevented induction of fecal bile acid excretion by GSK (Fig. 4A). After 4 weeks of treatment, analysis of the bile acid pool revealed that although all three treatments decreased tissue bile acid content and total bile acid pool, the GSK treatment and the AAV-FGF15 treatment reduced the total bile acid pool by ∼40% and ∼50%, respectively (Fisg. 4B and S2A). In comparison, the combined treatment reduced the total bile acid pool by ∼80% (Figs. 4B and S2A), which may be explained by the lack of hepatic CYP7A1 induction in the presence of GSK and maintained fecal bile acid excretion despite a smaller bile acid pool compared to the control mice (Fig. 4A–C). GSK reduced serum bile acid concentration by ∼50% and AAV-FGF15 and the combined treatment reduced serum bile acid concentration by ∼85% (Fig. 4D). Hepatic bile acid levels were significantly reduced by the combined treatment but were not affected by GSK or AAV-FGF15 (Fig. 4E). Analysis of gallbladder bile acid composition further revealed that GSK treatment decreased T-CDCA abundance from ∼70% to ∼40% and increased T-DCA abundance from ∼1% to ∼30% without affecting T-CA abundance compared to the untreated *Cyp2c70* KO mice (Figs. 4F and S2B). This could be partially explained by markedly increased hepatic CYP8B1 that shifted de novo bile acid synthesis toward CA production over CDCA production (Fig. 4G). However, since T-CA abundance was not increased by GSK, increased T-DCA abundance was also likely a result of simultaneous GSK inhibition of T-CA reuptake in the ileum, resulting in more T-CA conversion to deoxycholic acid (DCA) in the large intestine and subsequent transport of DCA to the liver (1). Sterol 27-hydroxylase (CYP27A1) and oxysterol and steroid 7α-hydroxylase (CYP7B1) mRNA treaded higher in the GSK-treated mice than controls (supplemental Fig. S2C, D), but increased expression of these genes in the alternative bile acid synthesis pathway did not correlate with increased T-CDCA abundance (Fig. 4F). Given that T-CDCA and T-DCA have very similar hydrophobicity index (19), these GSK-dependent bile acid composition changes did not markedly alter the hydrophobicity index of the gallbladder bile acids (Fig. 4H). Interestingly, the AAV-FGF15 treatment did not affect T-CA or T-DCA abundance but decreased T-CDCA abundance from ∼70% to ∼50% and increased T-UDCA abundance from ∼8% to ∼30% compared to the untreated *Cyp2c70* KO mice (Fig. 4F). This bile acid composition change reduced the hydrophobicity index of biliary bile acids (Fig. 4H), which may partially explain why only the AAV-FGF15 treatment, but not the GSK treatment, attenuated portal inflammation and ductular reaction although both treatments reduced the total bile acid pool by a similar magnitude. Although the combined treatment also increased hepatic CYP8B1 expression as the GSK treatment did, the bile acid composition changes resembled that of the AAV-FGF15 treatment group with ∼30% T-UDCA but only modestly increased T-DCA abundance (Fig. 4F, G), and reduced hydrophobicity index (Fig. 4H). The ∼80% reduction of total bile acid pool together with reduced bile acid hydrophobicity may contribute to the reversal of cholangiopathy and portal fibrosis in the combined treatment group. Lastly, we measured the mRNA of hepatic FXR and pregnane x receptor (PXR) target genes in the female mice. We found that the FXR target gene small heterodimer partner was only modestly increased in the female *Cyp2c70* KO mice compared to the WT mice, and its expression was not significantly affected by treatments (supplemental Fig. S2E). In contrast, the FXR target organic solute transporter β (OSTB) mRNA was strongly induced in the female *Cyp2c70* KO mice compared to the WT mice and was significantly reduced by AAV-FGF15 treatment and the combined treatment, and to less extent, the GSK treatment (supplemental Fig. S2F). The mRNA of PXR target genes cytochrome P450 3A11, multidrug resistance associated protein 3 and multidrug resistance associated protein 4 that are involved in bile acid metabolism and basolateral efflux was not induced in the female *Cyp2c70* KO compared to the WT mice or affected by various treatments (supplemental Fig. S2G–I). Hepatic sulfotransferase 2A1 (SULT2A1) was strongly repressed in the female *Cyp2c70* KO mice compared to the WT mice and was only partially restored in the treated female *Cyp2c70* KO mice (supplemental Fig. S2J). SULT2A1 is predominantly expressed at higher levels in female mouse livers (22), and bile acid accumulation in cholestasis has been shown to induce SULT2A1 via PXR activation (23, 24). The mechanisms underlying the strong repression of SULT2A1 in the female *Cyp2c70* KO mice are unclear. Taken together, the female *Cyp2c70* KO mice did not show robust transcriptional activation of hepatic FXR and PXR target genes, which were also minimally affected by treatments. This was possibly because the *Cyp2c70* KO mice mainly showed cholangiopathy but to less extent intrahepatocyte bile acid accumulation. In contrast, OSTB expression was mainly detected in mouse bile duct epithelial cells but not hepatocytes (25), which could explain more robust OSTB response to bile acid changes in the female *Cyp2c70* KO mice.

### The combined ASBT inhibitor and AAV-FGF15 treatment failed to improve cholangiopathy despite markedly reducing bile acid pool size in the male *Cyp2c70* KO mice

**Fig. 5:**
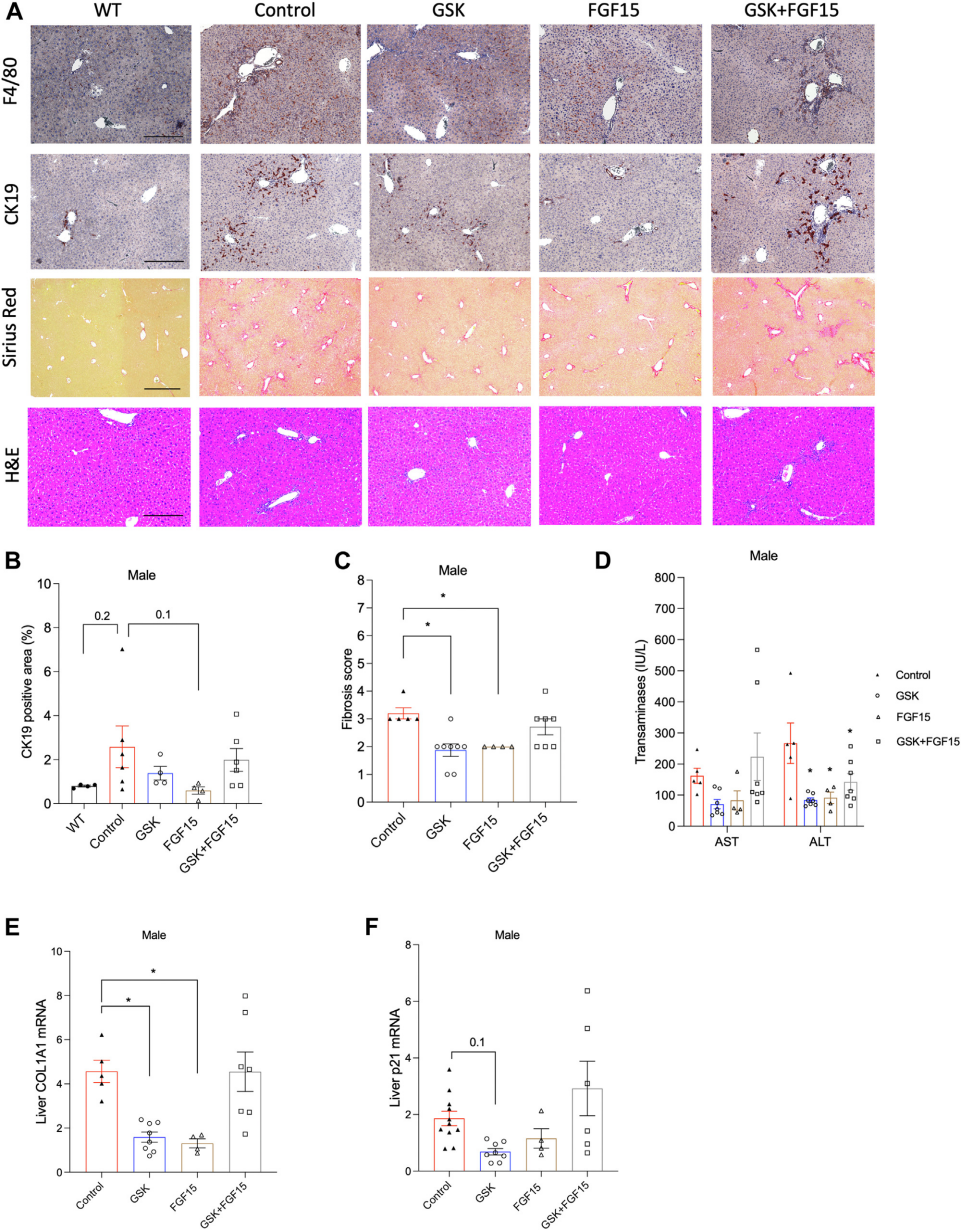
The therapeutic effects of the GSK, AAV-FGF15, and the combined treatment in the male *Cyp2c70* KO mice. Male *Cyp2c70* KO mice at 12 weeks of age were injected with AAV-FGF15 (1 × 10^11^ GC/mouse) indicated as “FGF15”. Some mice were treated with GSK (5 mg/kg/day) indicated as “GSK”. The *Cyp2c70* KO mice in the “Control” group and the “GSK” group were injected with AAV-Null (1 × 10^11^ GC/mouse). After 4 weeks, mice were fasted for 6 h from 9am to 3 pm and euthanized. A: Representative images of immunohistochemistry of F4/80 stain and CK19 stain, and Sirius Red and H&E stain. Scale bar = 250 um for F4/80, CK-19, and H&E; scale bar = 600 um for Sirius Red. Age matched WT mice were included for comparison. B: CK19 positive area per view was quantified with ImageJ. Software. N = 4–6. C: Ishak fibrosis score. N = 4–8. D: Serum alanine aminotransferase (ALT) and aspartate aminotransferase (AST). N = 4–7. E, F, Relative liver mRNA expression. N = 4–8. All results are expressed as mean ± SEM. “∗” indicates statistical significance (*P* < 0.05, one way ANOVA and Dunnett’s post hoc test), comparison is either versus “Control” or as indicated otherwise. FGF15, fibroblast growth factor 15; CK19, cytokeratin-19.

Likely because the male *Cyp2c70* KO mice developed much milder liver injury and portal fibrosis than the female *Cyp2c70* KO mice, we found that both the GSK treatment and the AAV-FGF15 treatment were able to attenuate portal inflammation, ductular reaction, and portal fibrosis in the male *Cyp2c70* KO mice (Fig. 5A–C). To our surprise, combining the two treatments failed to improve cholangiopathy or portal fibrosis in the male *Cyp2c70* KO mice (Fig. 5A–C). These histological changes were generally consistent with the changes of serum transaminases, hepatic mRNA expression of the fibrogenesis marker COL1A1, and the senescence marker p21 in these mice (Fig. 5D–F).

**Fig. 6:**
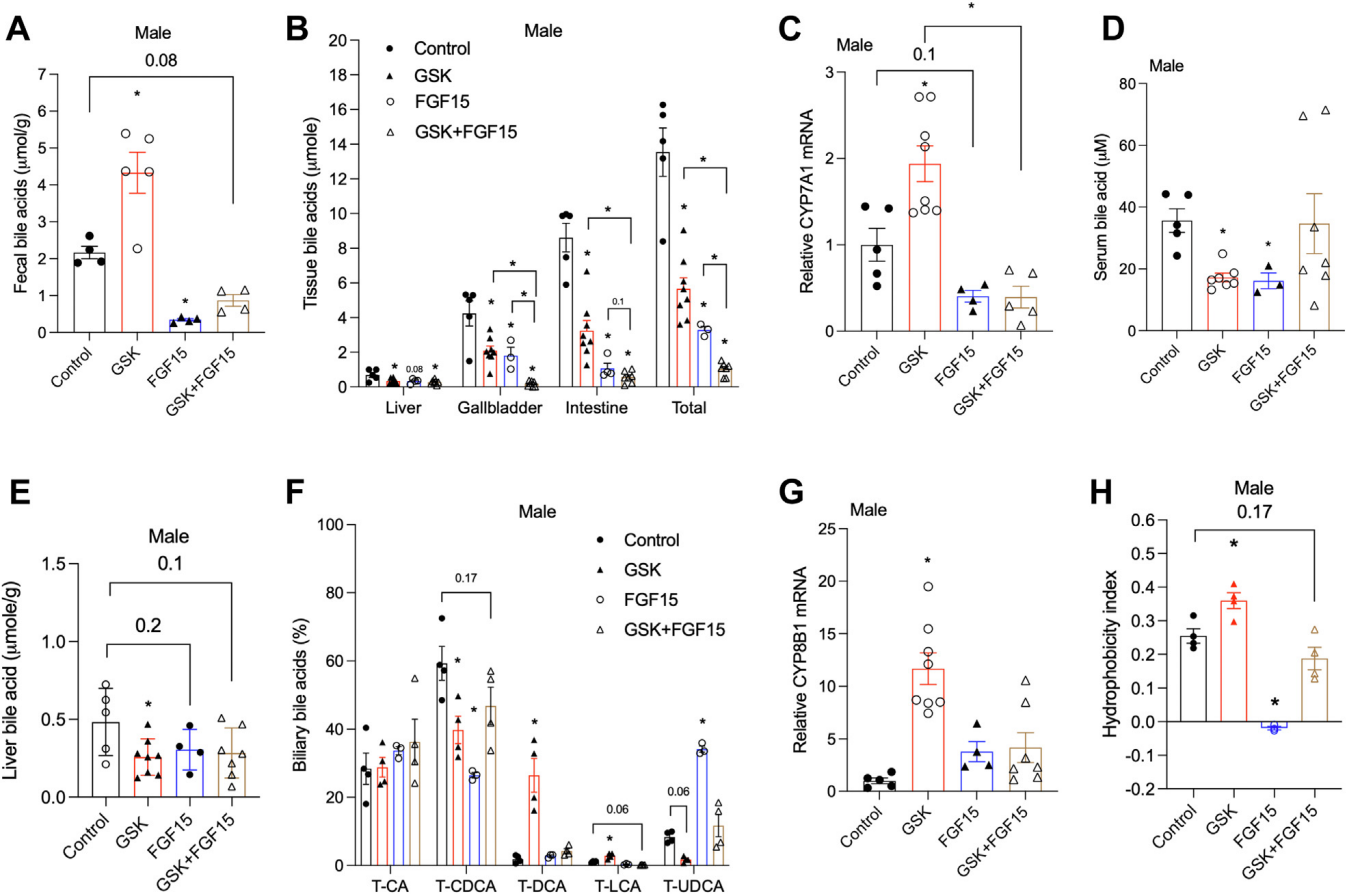
The Effects of GSK, AAV-FGF15, and the combined treatment on bile acid metabolism in the male *Cyp2c70* KO mice. Male *Cyp2c70* KO mice at 12 weeks of age were injected with AAV-FGF15 (1 × 10^11^ GC/mouse) indicated as “FGF15”. Some mice were treated with GSK (5 mg/kg/day) indicated as “GSK”. Mice in the “Control” group and the “GSK” group were injected with AAV-Null (1 × 10^11^ GC/mouse). After 4 weeks, mice were fasted for 6 h from 9am to 3 pm and euthanized. A: Fecal total bile acids after 2 weeks of various treatments. N = 4–5. B: Tissue total bile acid content and total bile acid pool. N = 3–8. C: Relative liver mRNA expression. N = 4–8. D: Serum bile acid concentration. N = 3–7. E: Liver total bile acid normalized to liver weight. N = 4–8. F: Gallbladder bile acid pool composition. N = 3–4. G: Relative liver mRNA expression. N = 4–8. H: Hydrophobicity index calculated based on gallbladder bile acid composition. N = 3–4. All results are expressed as mean ± SEM. “∗” indicates statistical significance (*P* < 0.05, one way ANOVA and Dunnett’s post hoc test), comparison is either versus “Control” or as indicated otherwise. FGF15, fibroblast growth factor 15.

Further analysis revealed that the GSK treatment increased fecal bile acid excretion while the AAV-FGF15 treatment and the combined treatment decreased fecal bile acid excretion after 2 weeks of treatment (Fig. 6A). After 4 weeks of treatment, the GSK treatment decreased bile acid pool size by ∼60%, the AAV-FGF15 treatment reduced bile acid pool size by ∼80%, while the combined treatment reduced the bile acid pool size by more than 90% in the male *Cyp2c70* KO mice (Figs. 6B and S3A). GSK treatment increased hepatic CYP7A1 mRNA, which was reduced in the AAV-FGF15 group and the combined treatment group (Fig. 6C). Serum bile acid concentration was lower in the GSK group and the AAV-FGF15 group (Fig. 6D). However, serum bile acid concentration was not reduced in the combined treatment group despite markedly smaller bile acid pool size (Fig. 6D). Liver bile acid levels were lower in all three treated groups than the control group (Fig. 6B, E). The GSK treatment and the AAV-FGF15 treatment resulted in similar bile acid pool composition changes in the male *Cyp2c70* KO mice as they did in the female *Cyp2c70* KO mice, with the GSK treatment increasing T-DCA abundance and the AAV-FGF15 treatment increasing T-UDCA abundance to ∼35% of the total bile acid pool (Figs. 6F and S3B). In the combined treatment group, there was only a trend toward reduced T-CDCA abundance and increased T-UDCA abundance but these changes were very modest and statistically insignificant (Figs. 6F and S3B). GSK treatment alone but not in the combined treatment increased hepatic CYP8B1 mRNA expression (Fig. 6G). Lack of improved liver injury could partially contribute to the lack of CYP8B1 mRNA increase and serum bile acid reduction in the male *Cyp2c70* KO mice under the combined treatment. Due to biliary bile acid composition changes, GSK treatment slightly but significantly increased the hydrophobicity index of biliary bile acids, the AAV-FGF15 treatment significantly reduced the hydrophobicity index of biliary bile acids and the combined treatment tended to lower the hydrophobicity index of biliary bile acids than the control group (Fig. 6H). Therefore, the combined treatment resulted in more than 90% reduction of the bile acid pool with a composition that was similar to that of the untreated male *Cyp2c70* mice but failed to improve cholangiopathy or portal fibrosis. Analysis of hepatic gene expression revealed that small heterodimer partner mRNA trended higher in the male *Cyp2c70* KO mice than the WT mice and was reduced by GSK treatment but not AAV-FGF15 or the combined treatment (supplemental Figs. S3C). OSTB mRNA was significantly higher in the male *Cyp2c70* KO mice and was reduced by all treatments (supplemental Figs. S3D). Similar to the female *Cyp2c70* KO mice, the male *Cyp2c70* KO mice showed only a trend toward higher expression of PXR targets cytochrome P450 3A11, multidrug resistance associated protein 3, multidrug resistance associated protein 4, or SULT2A1, which were not affected by various treatments (supplemental Fig. S3E–H).

### Restored gut barrier integrity by the combined treatment correlated with reduced colonic LCA exposure in the male and female *Cyp2c70* KO mice

**Fig. 7:**
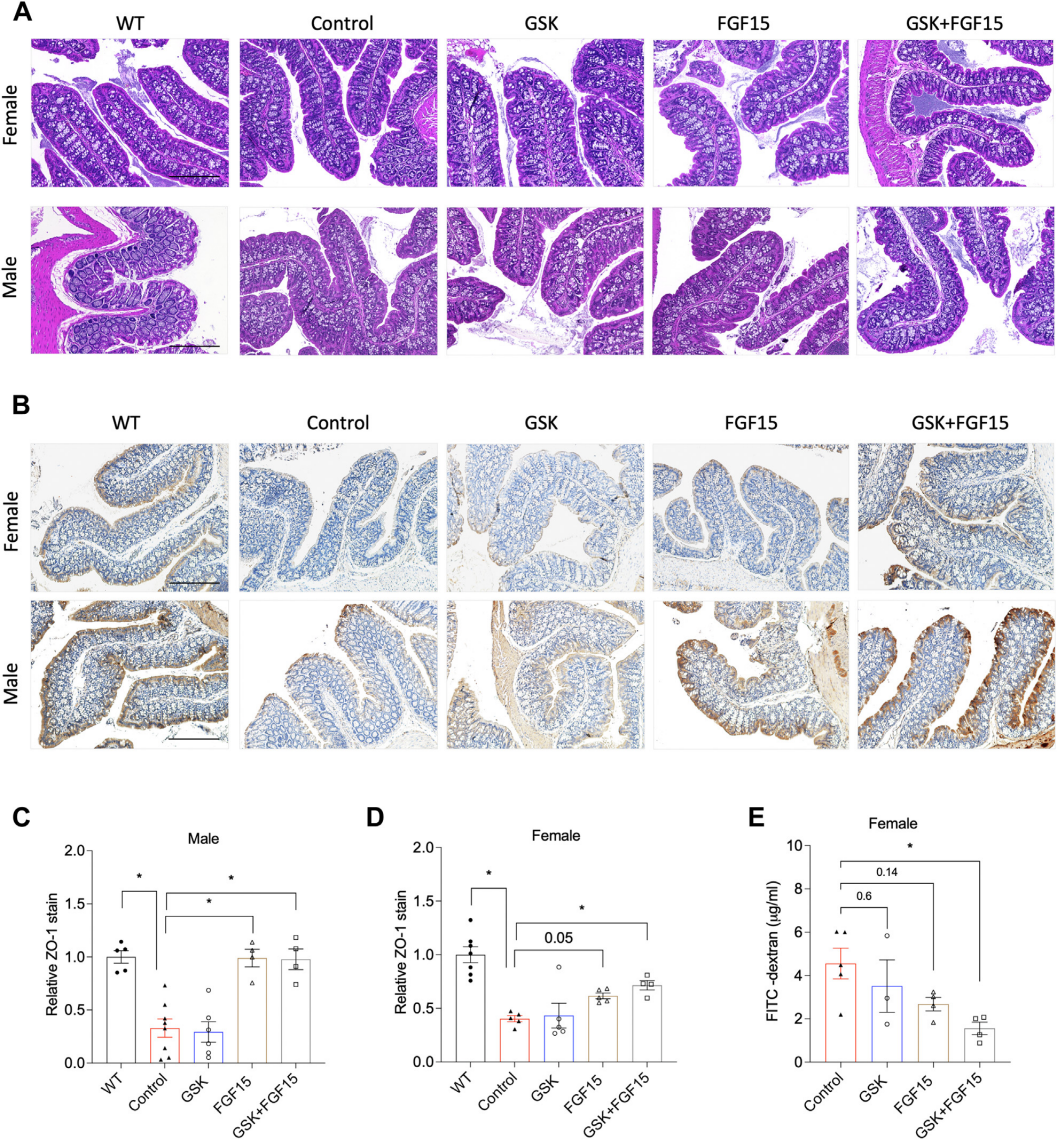
The combined treatment restored gut barrier integrity in the male and female *Cyp2c70* KO mice. Male and female *Cyp2c70* KO mice at 12 weeks of age were injected with AAV-FGF15 (1 × 10^11^ GC/mouse) indicated as “FGF15”. Some mice were treated with GSK (5 mg/kg/day) indicated as “GSK”. Mice in the “Control” group and the “GSK” group were injected with AAV-Null (1 × 10^11^ GC/mouse). After 4 weeks, mice were fasted for 6 h from 9 am-3 pm and euthanized. A: Representative images of colon H&E stain. B–D. Representative images of colon immunohistochemistry stain of ZO-1 (A). ZO-1 positive area was quantified with ImageJ software and normalized to villi area. N = 4–8. Scale bar = 250 um. E: Serum FITC-dextran concentration. N = 3–5. All results are expressed as mean ± SEM. “∗” indicates statistical significance (*P*<0.05, one way ANOVA and Dunnett’s post hoc test), comparison is either versus “Control” or as indicated otherwise. FGF15, fibroblast growth factor 15; ZO-1, Zonula Occludens-1.

Impaired gut barrier integrity in the *Cyp2c70* KO mice was reported recently (20). Bile acids are known to play important roles in maintaining normal gut functions under physiological condition, while exposure to high levels of hydrophobic bile acids under diseased conditions can also impair gut barrier function (26). It was recently shown that UDCA supplementation restored gut barrier integrity in the *Cyp2c70* KO mice (20), suggesting that impaired gut barrier integrity was associated with the hydrophobic bile acid exposure in the *Cyp2c70* KO mice. Because of GSK, AAV-FGF15 and the combined treatment are expected to have differential impact on colonic bile acid flux, we next investigated their effect on gut barrier integrity. Initial evaluation of H&E sections by a clinical pathologist did not reveal abnormal histological features of the colon epithelium of both male and female *Cyp2c70* KO mice, either untreated or treated with GSK, AAV-FGF15, or the combined treatment (Fig. 7A). However, immunohistochemistry staining of the tight junction protein Zonula Occludens-1 (ZO-1) revealed significantly reduced villus ZO-1 expression in the colon of both male and female *Cyp2c70* KO mice than the WT mice (Fig. 7B–D), consistent with previous report (20). Further evaluation of the treatment effects revealed that the GSK treatment did not affect ZO-1 level while the AAV-FGF15 treatment and the combined treatment increased ZO-1 levels in both male and female *Cyp2c70* KO mice compared to the control (Fig. 7B–D). Because increased ZO-1 suggests improved gut barrier function, we further investigated gut permeability to FITC-Dextran in female *Cyp2c70* KO mice under various treatment conditions. The results showed that the combined treatment, and to less extent the AAV-FGF15 treatment, but not the GSK treatment, decreased gut permeability to FITC-dextran compared to the untreated controls (Fig. 7E).

**Fig. 8:**
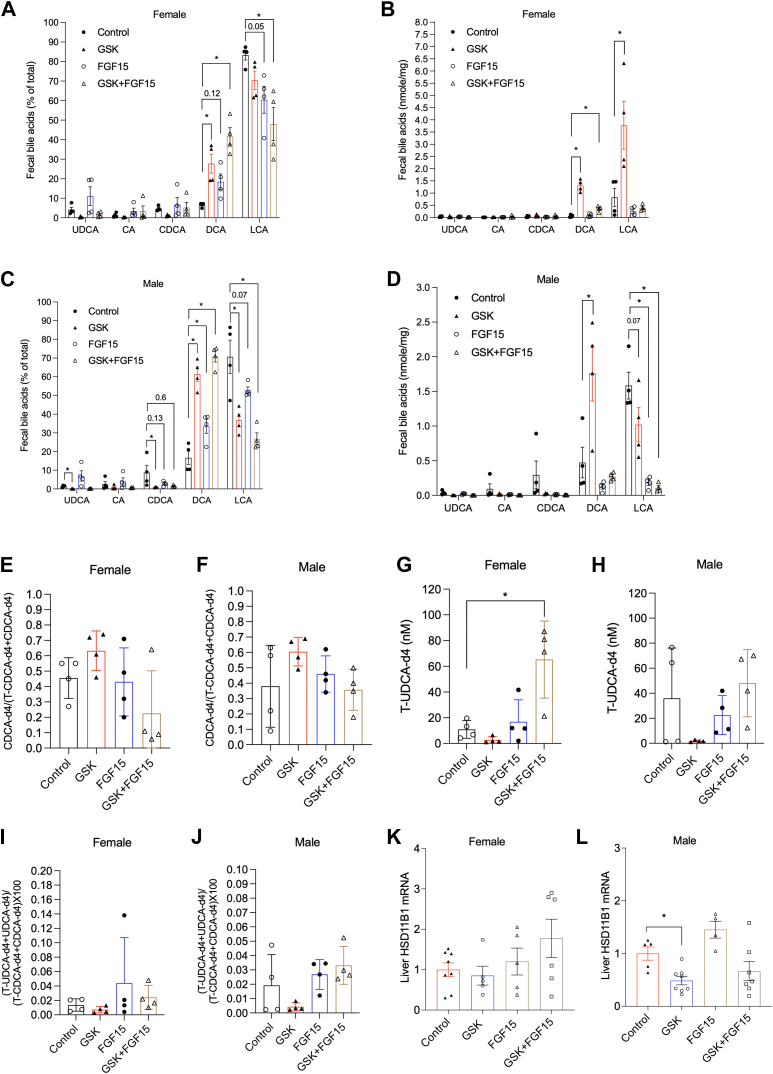
The combined treatment reduced fecal LCA abundance in the male and female *Cyp2c70* KO mice. Male and female *Cyp2c70* KO mice at 12 weeks of age were injected with AAV-FGF15 (1 × 10^11^ GC/mouse) indicated as “FGF15”. Some mice were treated with GSK (5 mg/kg/day) indicated as “GSK”. Mice in the “Control” group and the “GSK” group were injected with AAV-Null (1 × 10^11^ GC/mouse). After 4 weeks, fresh feces were collected from each individual mouse. A, C, Fecal bile acid composition. N = 4. B, D, Fecal bile acid amount normalized to fecal sample weight. N = 4. E–J. LC-MS measurement of T-CDCA-d4 transformation in fecal slurry as described in “Methods”. N = 4. E, F, The ratio of CDCD-d4 to the sum of T-CDCA-d4 and CDCA-d4. G, H, T-UDCA-d4 concentration measured in the fecal slurry extracts. I, J, The ratio of T-UDCA-d4 and UDCA-d4 to the sum of T-CDCA-d4 and CDCA-d4 expressed as percentage. K, L. Liver mRNA expression. N = 4–8. All results are expressed as mean ± SEM. “∗” indicates statistical significance (*P* < 0.05, one way ANOVA and Dunnett’s post hoc test), comparison is either versus “Control” or as indicated otherwise. CDCA, chenodeoxycholic acid; FGF15, fibroblast growth factor 15; LCA, lithocholic acid; T-CDCA, tauro-conjugated CDCA.

To understand how treatment-mediated alteration of bile acids may contribute to improved gut barrier integrity, we analyzed fecal bile acid composition, which has been shown to closely reflect colonic bile acid composition after microbial transformation of bile acids (16). Interestingly, we found that lithocholic acid (LCA) accounted for ∼ 85% of total fecal bile acids with very low abundance of DCA, UDCA, CA, and CDCA and largely undetectable conjugated bile acids in the untreated female *Cyp2c70* KO mice (Fig. 8A). The GSK treatment slightly reduced the LCA abundance and increased DCA abundance (Fig. 8A), which is consistent with our speculation that GSK treatment promotes DCA synthesis in the large intestine. In addition, the absolute amount of the fecal LCA and DCA were higher in the GSK-treated mice than the untreated mice due to increased overall fecal loss (Fig. 8B). In contrast, the fecal DCA abundance was higher while the fecal LCA abundance was lower in the AAV-FGF15 group and the combined treatment group than the untreated controls (Fig. 8A), suggesting reduced LCA exposure in the colon of these mice. These treatments also have very similar effects on fecal bile acids in the male *Cyp2c70* KO mice (Fig. 8C, D). Taken together, these data suggest that, unlike the colon of WT mice that was mainly exposed to DCA and the hydrophilic MCAs (27, 28, 29), the colon of the *Cyp2c70* KO mice was predominantly exposed to high levels of LCA and improved gut barrier integrity by the AAV-FGF15 treatment and the combined treatment closely correlated with reduced LCA exposure in the male and female *Cyp2c70* KO mice. GSK treatment did not reduce total colon bile acid exposure and did not improve gut barrier integrity.

### Treatment effects on gut microbial UDCA synthesis activity in the *Cyp2c70* KO mice

CDCA epimerization sequentially catalyzed by gut microbial 7α-hydroxysteroid dehydrogenase (7α-HSDH) and 7β-hydroxysteroid dehydrogenase (7β-HSDH) produces UDCA in humans and conventionally raised mice (30, 31, 32), while germ-free mice were also able to produce T-UDCA via mechanisms independent of gut microbiome (27). However, the fecal bile acid composition is markedly different from the hepatobiliary bile acid composition due to the microbial transformation of bile acids and poorly correlates with biliary bile acid composition (Fig. 4, Fig. 6, Fig. 8G). We also found that the fecal bile acid composition did not show consistent UDCA enrichment correlating with increased T-UDCA enrichment in the gallbladder bile of the AAV-FGF15–treated male and female mice and the combined treatment group of female mice (Fig. 4, Fig. 6, Fig. 8G). Therefore, we next incubated T-CDCA-d4 in fecal slurry from each group of mice and measured the production of CDCA-d4, UDCA-d4, and T-UDCA-d4 over a 6-h period that was similar to the colonic transit time in mice (33, 34). The ratio of CDCA-d4 to the sum of T-CDCA-d4 and CDCA-d4 suggests that, despite relatively large variations, ∼40–50% of the T-CDCA-d4 was deconjugated by bacterial bile salt hydrolase activity in the control groups and various treatments did not significantly alter T-CDCA-d4 deconjugation efficiency (Fig. 8E, F). The net production of T-UDCA-d4 only significantly increased in the fecal slurry of the combined treatment group of the female *Cyp2c70* KO mice (Fig. 8G, H). Interestingly, UDCA-d4 was not detected in any of the fecal slurry mixture despite that comparable levels of CDCA-d4 and T-CDCA-d4 were present (Fig. 8E, F), suggesting the possibility that T-CDCA-d4 was the preferred substrate for T-UDCA-d4 production under the in vitro assay condition (35, 36). It was estimated based on the T-UDCA-d4+UDCA-d4 to T-CDCA-d4+CDCA-d4 ratio that only less than 0.05% of the added T-CDCA-d4 was converted to T-UDCA-d4 (Fig. 8I, J). These data suggest that the gut microbial production of UDCA was very low and unlikely responsible for the observed biliary T-UDCA enrichment in the AAV-FGF15 groups and the combined treatment groups. Lastly, we measured the mRNA of hepatic 11β hydroxysteroid dehydrogenase-1 (HSD11B1), which has been shown to convert the LCA metabolite 7-oxolithocholic acid mainly to CDCA and, to much less extent, UDCA (37). We found that hepatic HSD11B1 mRNA was not induced in the AAV-FGF15 groups or the combined treatment groups (Fig. 8K, L).

## DISCUSSION

Human patients with cholestasis may benefit from reduction of bile acid pool size and hydrophobicity, which serves as a key rationale for developing and testing several bile acid-based therapeutics for cholestasis treatment (6). Currently, most studies in experimental rodent models and human clinical trials have mainly focused on bile acid-based monotherapies (7, 8, 9, 10, 11, 38, 39, 40), while whether combining existing therapies with distinct mechanisms of action can potentially achieve improved therapeutic efficacy has not been well studied. Furthermore, commonly used rodent experimental cholestasis models have a different bile acid pool composition than that of humans (18). In this study, we have compared and contrasted the effects of the gut-restricted ASBT inhibitor GSK, the AAV-FGF15 treatment that mimics the effect of an FGF19 analogue, and the combined treatment on bile acid metabolism and cholangiopathy and portal fibrosis induced by a human-like hydrophobic bile acid pool in the *Cyp2c70* KO mice. Major findings revealed by this study are discussed below.

In both male and female *Cyp2c70* KO mice, we demonstrated that the combined treatment was able to achieve a significantly higher degree of bile acid pool reduction than either single treatment. De novo bile acid synthesis and ileal bile acid uptake are two bile acid-sensing mechanisms that help maintain a relatively constant bile acid pool under normal physiology. However, bile acid synthesis has not been reported to be consistently repressed in all cholestatic conditions in humans and experimental models (3, 41, 42). Reduced bile acid flow into the intestine in cholestasis may diminish both the intestinal FGF15/19-mediated signaling and fecal bile acid excretion, explaining why the adaptive responses are mostly insufficient to significantly limit bile acid toxicity in cholestasis. In cholestasis patients with already repressed hepatic bile acid synthesis, therapeutics-targeting de novo bile acid synthesis may provide little additional benefits. Because the combined treatment simultaneously limited hepatic bile acid synthesis and intestinal bile acid reuptake, it achieved a much stronger reduction of bile acid pool than either single treatment. In the female *Cyp2c70* KO mice exhibiting severe progressive cholangiopathy and portal fibrosis and thus representing a difficult to treat cholestasis condition, additional reduction of the bile acid pool was likely a major mechanism that contributed to the reversal of cholangiopathy and portal fibrosis by the combined treatment. In contrast, the GSK treatment failed to offer therapeutic benefits, while the AAV-FGF15 treatment only alleviated cholangiopathy but not portal fibrosis in the female *Cyp2c70* KO mice. A recent study reported that OCA single treatment for 4 weeks failed to alleviate cholangiopathy or portal fibrosis when the OCA treatment was initiated in 12 weeks old female *Cyp2c70* KO mice (43). Another recent study reported that treating 4 weeks old female *Cyp2c70* KO mice with an ASBT inhibitor SC-435 for 8 weeks fully prevented cholangiopathy and fibrosis (17). These findings collectively suggest that liver cholangiopathy and portal fibrosis become more difficult to reverse with interventions as they progress rapidly with age in the female *Cyp2c70* KO mice. This is consistent with our findings that GSK and AAV-FGF15 single treatment were sufficient to alleviate cholangiopathy and portal fibrosis in the male 12 weeks old *Cyp2c70* KO mice with much milder liver pathology than the female 12 weeks old *Cyp2c70* KO mice.

However, the lack of protection by the combined treatment in the male *Cyp2c70* KO mice is puzzling and not well understood. We think that it is unlikely a result of the strong reduction of overall bile acid pool per se, because the combined treatment caused a similar degree of bile acid pool reduction in the female *Cyp2c70* KO mice that was associated with improved liver pathology. One notable difference was that the female *Cyp2c70* KO mice progressively developed severe cholangiopathy and portal fibrosis with age, while the liver pathology in the male *Cyp2c70* KO mice was mild and self-limiting (20), although both male and female *Cyp2c70* KO mice were exposed to a bile acid pool of similar size and hydrophobicity. This suggests that the host sex-dependent factors play important roles in modulating the hydrophobic bile acid-driven cholangiopathy development and the differential responses to treatments, which remains to be investigated in the future. In humans, the opposite effect of ASBT inhibitor and FGF19 analogue treatment on hepatic bile acid synthesis rate has been confirmed by measurement of serum C4, a surrogate marker for de novo bile acid synthesis rate, but the actual magnitude of reduction in hepatic bile acid exposure and total bile acid pool by either single treatment is difficult to estimate accurately in humans (9, 44). However, it is reasonable to speculate that combining the two treatments could theoretically result in a stronger reduction of total bile acid pool and hepatic bile acid burden than either single treatment in humans.

Another finding of this study was that the AAV-FGF15 treatment and the combined treatment caused T-UDCA enrichment to ∼30% of the gallbladder bile acid pool in the female *Cyp2c70* KO mice. This effect was observed in the AAV-FGF15–treated male *Cyp2c70* KO mice but not in the combined treatment group of male *Cyp2c70* KO mice. It should be noted that biliary T-UDCA enrichment was not previously discovered in WT mice treated with AAV-FGF19 or the FGF15 transgenic mice (45, 46), suggesting that high level of T-CDCA in the human-like bile acid pool was required for the AAV-FGF15 treatment to promote T-UDCA enrichment in the *Cyp2c70* KO mice (32). Consistently, feeding WT mice CDCA only increased T-UDCA abundance to ∼5% of the total bile acid pool because the exogenously administered CDCA was efficiently converted to MCAs by CYP2C70 in WT mice (14, 47). A recent report showed that UDCA supplementation reversed cholangiopathy in *Cyp2c70* KO mice (20), which supported the protective effect of T-UDCA enrichment in the *Cyp2c70* KO mice. Under AAV-FGF15 treatment, other secondary bile acids T-DCA and T-LCA were not enriched. These potentially point to a treatment-induced shift of gut microbiome with enhanced enzyme activity for CDCA conversion to UDCA. However, our in vitro study in fecal slurry mixtures suggests that the overall microbial enzyme activity–mediated T-UDCA production has a very low efficiency and the evidence from these experiments do not suggest that gut microbial UDCA production may account for the biliary T-UDCA enrichment. It was recently reported that the gut microbiome was altered in the *Cyp2c70* KO mice compared to their WT littermates (20) but the subsequent pathophysiological impacts remain to be determined. Regarding microbial T-UDCA synthesis, a number of studies have found that different strains of bacteria are capable to synthesize UDCA from CDCA (32, 48, 49, 50, 51, 52, 53). However, further establishing a causative link between gut microbiome changes and UDCA synthesis, activity is hindered by the lack of a complete knowledge of the full list of gut bacteria strains harboring 7α-HSDH and 7β-HSDH enzyme activity, the relative level of the enzyme activities in different strains and how the 7α-HSDH and 7β-HSDH expression and activities are regulated in different strains in response to changes of gut bile acids (54). Without such knowledge, further analysis of gut microbiome may offer little insights in this regard and was therefore not pursued at presence. Mice can produce UDCA via microbial enzyme-independent mechanisms that are still poorly defined (27). Recent evidence suggests that HSD11B1 mediates hepatic UDCA synthesis (37) m = 8jm 0 but we found that its expression was not significantly affected by various treatments in the *Cyp2c70* KO mice. Analysis of the absolute amount of biliary bile acids (supplemental Figs. S2B and S3B) leads us to speculate that the AAV-FGF15 treatment preferentially reduced other biliary bile acid species but not T-UDCA, which could enrich T-UDCA and decrease hydrophobicity index without stimulating UDCA synthesis. In the combined treatment group, the marked reduction of total bile acid pool is expected to have a more significant pathological impact than modestly reduced bile acid hydrophobicity. Whether FGF19 analogue also promotes UDCA enrichment in human bile acid pool remains to be determined. A technical limitation is that measurement of the bile acid composition in humans is largely limited to using systemic blood samples or fecal samples and the results do not closely reflect the bile acid composition in the enterohepatic circulation.

It is considered that the *Cyp2c70* KO mice showed a “human-like” hydrophobic bile acid pool, but their biliary bile acid composition still differed from a typical human bile acid pool composition in that it consisted of relatively low abundance of T-CA and only trace amount of T-DCA (1). It should be noted that CYP8B1 was strongly repressed in the *Cyp2c70* KO mice compared to the WT mice, rendering a lower CA synthesis in the *Cyp2c70* KO mice than the WT mice. In contrast to the AAV-FGF15–mediated bile acid pool composition shift from T-CDCA to T-UDCA, the GSK treatment induced a shift from T-CDCA to T-DCA in the *Cyp2c70* KO mice. This could be partially due to a strong induction of CYP8B1, which increased hepatic CA synthesis. Importantly, GSK treatment resulted in increased T-CA in the colon where it is converted to DCA that is subsequently transported to the liver for conjugation. Indeed, the GSK-treated *Cyp2c70* KO mice showed a bile acid pool consisting of ∼30% T-DCA and ∼30–40% of T-CA and T-CDCA, which more closely resembled the human bile acid pool composition (1). GSK treatment is also expected to cause more T-CDCA to reach the colon where CDCA can be converted to LCA. However, microbial CDCA conversion to LCA is expected to be of low efficiency and gut LCA is more efficiently detoxified and excreted into feces. As a result, the fecal bile acid pool of the GSK-treated *Cyp2c70* KO mice contained mainly LCA and DCA and thus more closely resembled the human fecal bile acid composition (55, 56). Therefore, the strong repression of CYP8B1 underlies some key differences in the bile acid composition of the *Cyp2c70* KO mice from that of humans. It has been shown that deletion of CYP2A12, which converts DCA to CA and LCA to CDCA via 7α-rehydroxylation reaction in mice, can increase the relative biliary T-DCA abundance in the *Cyp2c70* KO mice, but CYP2A12 deletion also increased T-LCA abundance and decreased T-CA abundance in the bile (16). In the female *Cyp2c70* KO mice, GSK was the least effective treatment in reducing hepatobiliary bile acid levels, possibly due to the compensatory induction of hepatic CYP7A1 expression and bile acid synthesis. Furthermore, the resulting shift from T-CDCA to T-DCA in the GSK-treated *Cyp2c70* KO mice did not reduce bile acid pool hydrophobicity as the AAV-FGF15 treatment did. These may partially explain why GSK was not effective in alleviating severe cholangiopathy in the female *Cyp2c70* KO mice.

Reduced ZO-1 staining in both male and female *Cyp2c70* KO mice compared to WT mice suggested significantly impaired gut barrier integrity, which has also been reported by another group (20). Cholestasis has been associated with impaired gut barrier function, which in turn contributes to liver inflammation via the gut-liver axis (57, 58). Therefore, changes in the gut barrier integrity may also contribute to liver injury in the *Cyp2c70* KO mice. Interestingly, in contrast to the hepatobiliary exposure to T-CDCA–enriched bile acid pool, the colon of the *Cyp2c70* KO mice was predominantly exposed to the toxic LCA and to much less extent DCA, which correlated with higher abundance of T-CDCA in the bile of these mice. This was different from WT mice with DCA and the hydrophilic MCA being the major bile acids in the fecal samples (27, 28, 29). UDCA treatment has been shown to improve gut barrier function in the *Cyp2c70* KO mice (20), suggesting that hydrophobic bile acid exposure underlies gut barrier impairment in the *Cyp2c70* KO mice. These results collectively suggest that the protective effect of the combined treatment, and to a lesser extent the AAV-FGF15 treatment, against gut barrier impairment in the *Cyp2c70* KO mice may be mainly attributed to reduced hydrophobic bile acid flux through the colon. In contrast, GSK treatment promotes colon bile acid flux and did not improve gut barrier function in the *Cyp2c70* KO mice. Future studies are needed to investigate if the combined treatment could offer therapeutic benefits in experimental models of certain gut epithelial disorders where hydrophobic bile acids act as pathological drivers (26).

In summary, this study conducted in the *Cyp2c70* KO mice revealed that combining an ASBT inhibitor GSK with AAV-FGF15 treatment can achieve a higher magnitude of reduction of bile acid pool accompanied with increased hepatobiliary T-UDCA enrichment that lowered bile acid pool hydrophobicity. This combined treatment was highly effective in alleviating cholangiopathy and portal fibrosis in the female *Cyp2c70* KO mice, suggesting that combining ASBT inhibitor and FGF19 analogue could provide improved therapeutic efficacy in certain forms of cholestasis that could theoretically benefit from a much higher degree of bile acid pool reduction. The finding shows that the beneficial effect of the combined treatment was absent in the male *Cyp2c70* KO mice is quite puzzling and requires better mechanistic investigation in the future. In addition, whether the sex-dependent outcomes in response to the combined treatment is only limited to the Cyp2c70 KO mouse models or could also exist in humans remains to be answered.

## Data availability

All data, analytic methods, and study materials will be available to researchers upon request to the corresponding author.

## Supplemental data

This article contains supplemental data.

## Conflict of interest

The author declares that they have no conflicts of interest with the contents of this article.
