# Supplementary material for: Combining ASBT inhibitor and FGF15 treatments enhances therapeutic efficacy against cholangiopathy in female but not male Cyp2c70 KO mice
Source: J Lipid Res. 2023 Feb 3;64(3):100340. doi: 10.1016/j.jlr.2023.100340 (PMC9986646; doi:10.1016/j.jlr.2023.100340)
Supplement: Supplemental data [file mmc1.pdf]

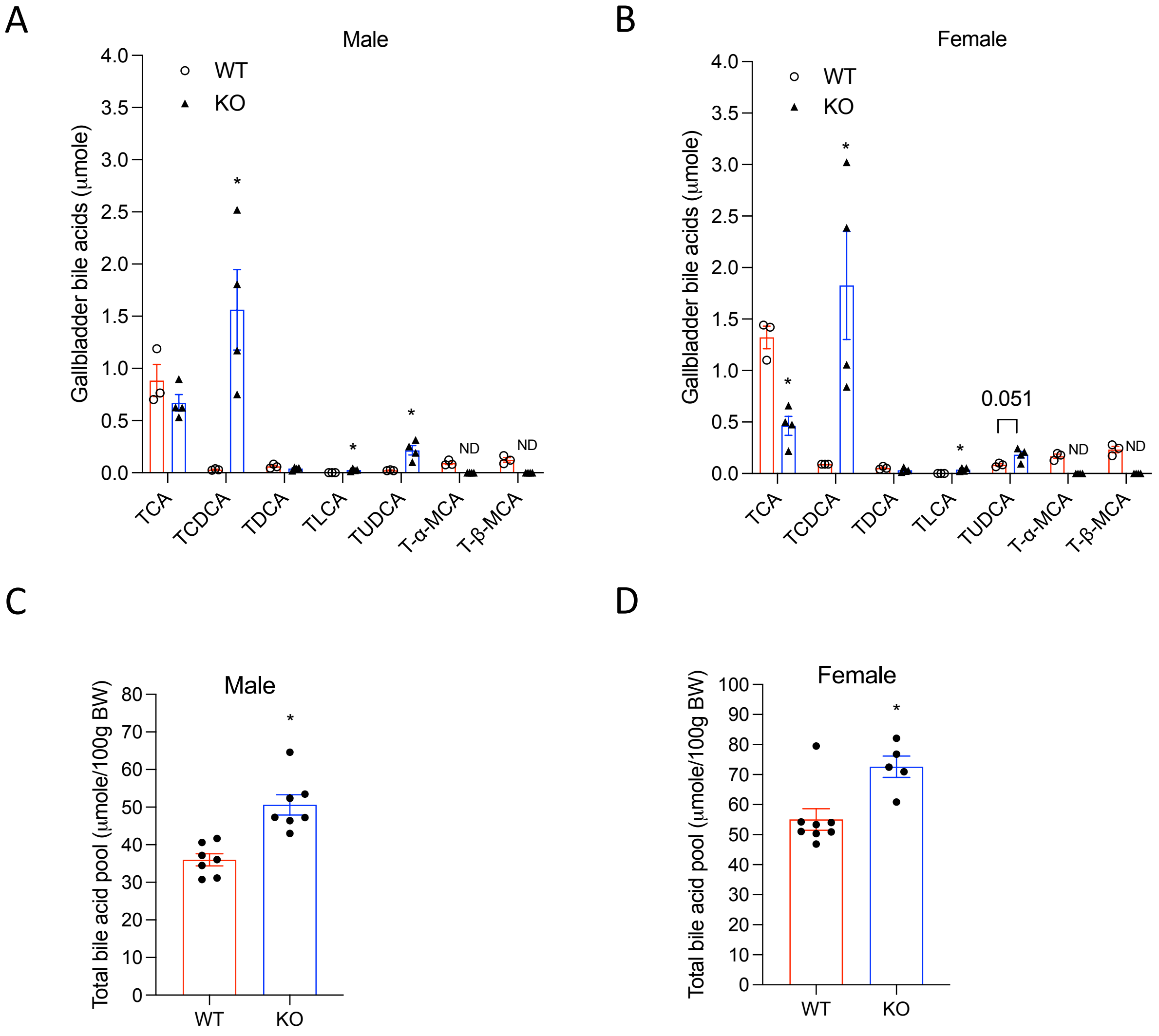

Supplementary Figure 1. The *Cyp2c70* KO mice show enlarged hydrophobic bile acid pool than WT mice. Male and Female WT and *Cyp2c70* KO mice at 16 weeks of age were fasted for 6 h from 9 am to 3 pm and euthanized. A, B. Gallbladder bile acid pool amount. n=3-4. C, D. Total bile acid pool normalized to body weight. n=5-8. All results are expressed as mean ± SEM. “*” indicates statistical significance ($p<0.05$, Student’s t-test), vs. WT. ND: not detectable.

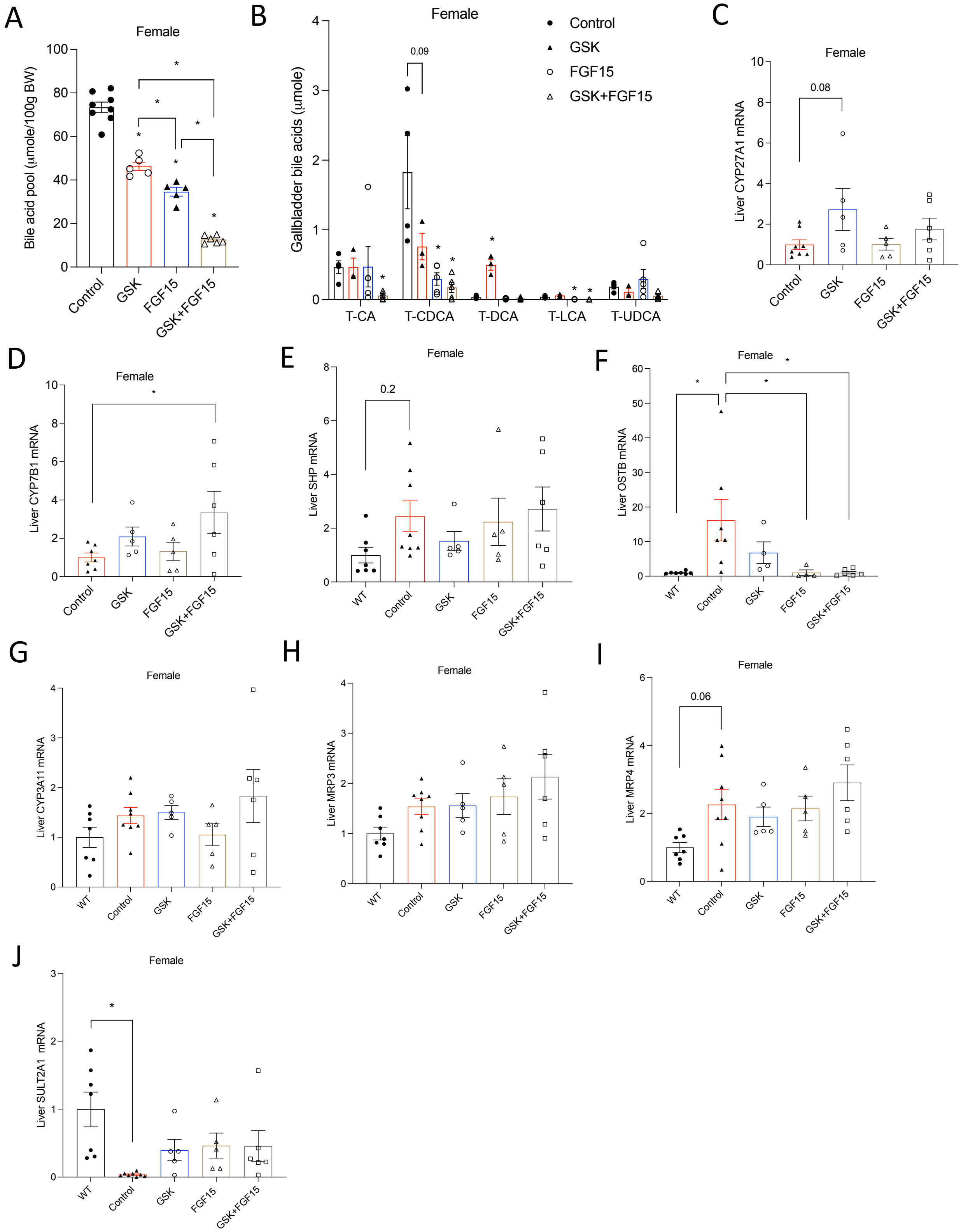

Supplementary Figure 2. The effects of GSK, AAV-FGF15, and GSK+AAV-FGF15 treatment on bile acid metabolism in the female *Cyp2c70* KO mice. Female *Cyp2c70* KO mice at 12 weeks of age were injected with AAV-FGF15 (1 x 1011 GC/mouse) indicated as "FGF15". Some mice were treated with GSK (5 mg/kg/day) indicated as "GSK". Mice in the "Control" group and the "GSK" group were injected with AAV-Null (1 x 1011 GC/mouse). After 4 weeks, mice were fasted for 6 h from 9am-3 pm and euthanized. A. Bile acid pool normalized to body weight. n=5-8. B. Gallbladder bile acid amount. n= 3-5. C-J. Relative liver mRNA expression. n=4-8. All results are expressed as mean ± SEM. "*" indicates statistical significance ($p<0.05$, one way ANOVA and Dunnett's post hoc test), comparison is either vs. "Control" or as indicated.

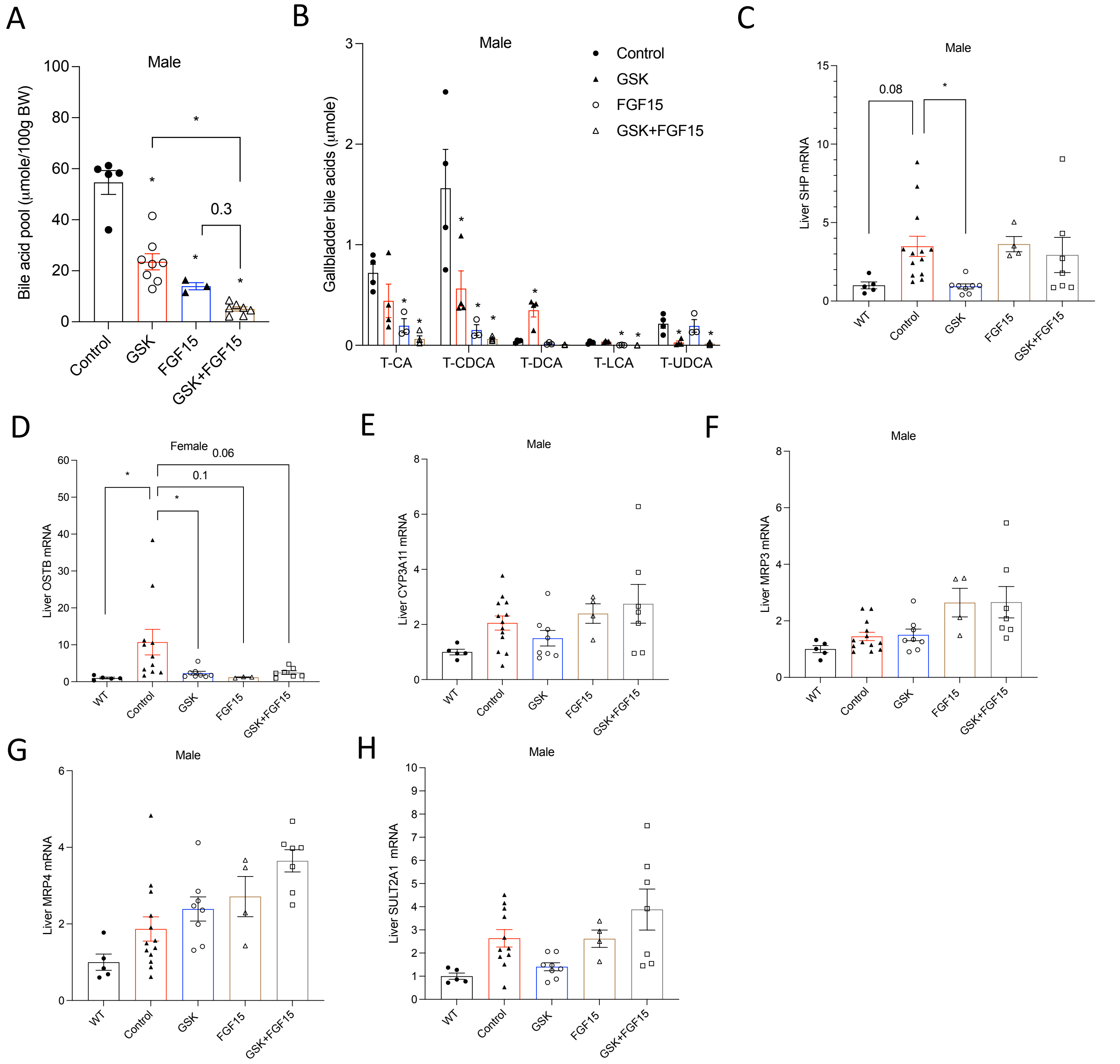

Supplementary Figure 3. The effects of GSK, AAV-FGF15, and GSK+AAV-FGF15 treatment on bile acid metabolism in the male *Cyp2c70* KO mice. Male *Cyp2c70* KO mice at 12 weeks of age were injected with AAV-FGF15 (1 x 1011 GC/mouse) indicated as "FGF15". Some mice were treated with GSK (5 mg/kg/day) indicated as "GSK". Mice in the "Control" group and the "GSK" group were injected with AAV-Null (1 x 1011 GC/mouse). After 4 weeks, mice were fasted for 6 h from 9am-3 pm and euthanized. A. Bile acid pool normalized to body weight. n=3-8. B. Gallbladder bile acid amount. n= 3-4. C-H. Relative liver mRNA expression. n=4-13. All results are expressed as mean ± SEM. "*" indicates statistical significance ($p<0.05$, one way ANOVA and Dunnett's post hoc test), comparison is either vs. "Control" or as indicated.

Supplementary Table 1: LC-MS bile acid detection parameters

Compound	Retention Time (min)	RT Window (min)	Precursor (m/z)	Product (m/z)	Collision Energy (V)
TUDCA	5.9	2	498.4	124.1	54
TUDCA-d4	5.9	2	502.4	80	56
TUDCA-d4	5.9	2	502.4	502.4	8
UDCA	8.9	2	391.3	391.3	8
UDCA-d4	8.9	2	395.3	395.3	8
CA	10.1	2	407.3	407.3	8
GCDCA-d4	10.1	2	452.5	452.5	8
TCDCA	10.2	2	498.4	124.1	54
TCDCA-d4	10.2	2	502.4	502.4	8
CDCA	12.9	2	391.3	391.3	8
CDCA-d4	12.9	2	395.4	395.4	8
DCA	13.4	2	391.3	391.3	8
LCA	15.9	2	375.3	375.3	8
TCA	7.4	2	514.4	124.1	55
TLCA	14.6	2	482.4	124.1	52
TDCA	11.2	2	498.4	124.1	57
T-α-MCA	3.9	2	514.2	514.2	8
T-β-MCA	4.1	2	514.2	514.2	8
